# Effect of family linked factors on sibling's oral health

**DOI:** 10.6026/973206300200079

**Published:** 2024-01-31

**Authors:** A Pravin Kumar, K Palaiyatharasi, K Ponni, N Smitha, J Vikraman, M Vennila

**Affiliations:** 1 Government Arignar Anna Memorial Cancer Hospital & Research Institute, Tamilnadu, India; 2 Government Namakkal medical college & Hospital, Tamilnadu, India; 3 Government Chengalpattu Medical College and Hospital, Tamilnadu, India; 4 Government Medical College & ESIC Hospital Coimbatore, Tamilnadu, India; 5 Government Villupuram Medical College and Hospital, Tamilnadu, India

**Keywords:** Sibling dental caries, oral health problem, family linked factors

## Abstract

The association between family factors and dental caries/dental plaque development in children is of interest. The parents of pediatric age group seeking treatment from the Government Chengalpattu Medical college hospital, who accompanied with two or more siblings, were included. A questionnaire was given to gather data on the parents' socioeconomic status and family size. The children underwent clinical examination to evaluate the state of their oral health. Results showed a significant relationship between dental plaque and caries experience of child with gender, parents' education, occupation and socioeconomic status. So we conclude that the family related factors such as parent's education, occupation, income and SES exerted a definite impact in the oral health of children than the family size and sibling factors.

## Background:

Oral health is an essential component of general health [1], A child's growth and cognitive development are also greatly impacted by poor oral health because it can hinder school participation, nutrition, and concentration [2]. Parents have a direct responsibility for their children's dental health and can play a major role in preventing oral diseases in children [3]. Dental Caries has long been understood to be arises mainly from long-term interactions between fermentable dietary carbohydrates and cariogenic oral micro-biota in a susceptible host [4]. However, this perception of caries causation ignores a number of other significant Family-level variables such as parents' socioeconomic status, Occupation, Education, etc. Observations of association between siblings in the prevalence of dental caries have long been an area of interest for dental researchers. In 1959 Mansbridge's [5] in a study concluded that environmental factors undoubtedly have greater impact but genetic factors also play a role in the development of dental caries. In 2010, a review by Shearer and Thomson [6] , discuss different mechanisms by which parents influence their children's oral health. The recent literature clearly shows that the family has an important role in determining children's oral health [7]. Therefore, it is of interest to estimate the association in Dental caries/Dental Plaque between siblings, and to investigate whether this varied according to the family level factors such as parents' education, occupation, family income, socioeconomic status and family size.

## Methodology:

This cross-sectional study was conducted using a convenience sample of parents who accompanied their children (2 or more siblings), seeking general treatment from Government Chengalpattu Medical College & Hospital (GCMCH) from March 2023 to June 2023. Children aged from 2 to 14 years whose parents agreed to participate in the study were included. Parents and Children affected by Congenital and developmental anomaly were excluded. Prior to the start of the study, a self-administered questionnaire was given to the parents of pediatric age group who accompanied with their child. The pre-printed forms in regional language were disturbed to the parents and their filled data was collected. The questions were explained in the patient's native language for those who were illiterate, and their answers were recorded. A questionnaire was given, to gather information on the parents' socioeconomic status (as measured by the Kuppuswamy socioeconomic scale [8]), age and family size. For Dental caries assessment, the numbers of decayed, filled and missing teeth (deft and DMFT) were noted for children with caries. The deft/DMFT was determined based on WHO Oral Health Survey methods [9] , and the Oral hygiene status was evaluated using Plaque index (PLi). The PLi was determined based on the WHO Oral Health Survey methods [9]. For Nonparametric statistical purposes, caries & plaque will be classified as present or absent. The study was approved by the Institutional Ethics Committee. All participants provided written informed consent before participating in the study

## Results:

Overall, from 108 parents, 232 children aged 2 -14 years (average age 7 years) old were involved in this study, of whom 106 children (45.7%) were males, and 126 (54.3%) were females. A total of 141 children (60.7%) had caries of which 53(22.8%) were male and 88 (37.9%) were females. A total of 91 children (39.2%) were caries free of which 53 (22.8%) were male children. A total of 216 (93.1%) children had no missed component and none of the children had a Dental filling. The DMFT/deft score was zero for 89 (38.4 %) children of which 51 (22%) were male and 38 (16.4%) were female children. The Plaque score was zero for 120 (51.7 %) children of which 68 (29.3%)were male and 52 were(22.4%) female children. A dental plaque score 1, 2, and 3 for 82 (35.3%), 12 (5.2%), and 18 (7.8%) children respectively was recorded. Their parents age ranged from 27 to 49 years, among whom 2.6 % were illiterate, 6.9 % graduates, 27.6 % were diploma holders, 7.8 %, 31.0 %, and 24.1 % had completed primary, middle and High school respectively. When stratified by occupation, 32.8 % were Machine operators / Assemblers, 15.5 % Clerks, 15.5 % Skilled worker/ shop market sales workers, 13.8% elementary occupation, 12.1% skilled agricultural/fishery workers and rest all 10.3% were craft, trade, technician and associated professionals. When segregated based on salary, majority (60.3%), had family income in rupees of Rs 9232 to Rs 27648 per month, 25.9% had salary of Rs 27654 to 45089 per month, 10.3% had salary less than Rs 9226 per month, and 3.2% had salary of Rs 46095 to 68961 per month.

As per Kuppuswamy [8] socio-economic status scale 2022 , 50.9%, 31.9%, 10.3%, and 6.9% belongs to upper lower, lower middle, upper and upper middle class respectively. In family size, 74.1% belong to nuclear family and rest 25.9 % had family members of five and above. A chi-square test of independence was performed to examine the relation between gender and dental caries / plaque. For statistical purpose, DMFT/deft Score 0 or 1 & above and for plaque, whether present or absent alone was taken into consideration. The relation between the gender and DMFT/deft was significant, X2 (1, N = 232) = 7.85, p = .005. The relation between the gender and plaque was very significant, X2 (1, N = 232) = 12.07, p = .001. Table 1 show the results of A bivariate analysis which was carried out to check for the association between each independent variable and the dental caries/ plaque status of the child. Variables showing a significant association were then subjected to a logistic regression analysis. Table 1 show the comparative statistic for DMFT/deft and Dental plaque score in children between different variables of respondents.

## Socio-demographic factor analysis:

On evaluating the SES variables using Kuppuswamy's SES Scale, we found that 50.9% of the children were from the upper lower class strata of society. A stepwise logistic regression found parents level of education (P < 0.006), Occupation (P < 0.000) & SES (P < 0.007) to be significantly associated with the dental caries status of children. Within education level -Illiterate parents (P < 0.002), and among different Occupation, Craft & related trade workers (P < 0.002), Machine operators / assemblers (P < 0.000) were significantly related to increased prevalence of dental caries. Among different SES scale, upper middle class was significantly related to (increased incidence of caries) - presence of caries (P < 0.002) [Table 1]. No significant association was seen with the family income & size (nuclear/ join family). sBinary logical regression reveals a statistically significant relation between presence of dental plaque with a Parents Education (P < 0.004), Occupation (P < 0.000), Family income (P < 0.000) and SES (P < 0.047). Within the above significant relation groups, illiterate parents (P < 0.010), Clerk (P < 0.000) & Skilled workers/ Agricultural/ Market sales (P < 0.000), family income except less than Rs 9226 group and lower middle & upper lower SES group are significant predictor of presence of dental plaque.

## Sibling factor analysis:

The mean & standard deviation values of DMFT/deft, Plaque scores in the participants of first and second /subsequent child is shown in Table 2. An independent-sample t-test was conducted to compare the DMFT and Plaque Index for first and second/subsequent child. There was no statistically significant difference in the score for first child (M = 2.64, SD =3.55) and second/subsequent child (M = 3.27, SD =3.99) Conditions; t (230) = 1.26, p = 0.21' for DMFT/deft and similarly there was no statistically significant difference between the first child (M = 0.74, SD =0.94) and second/subsequent child (M = 0.65, SD =0.84) Conditions; t (230) = 0.82, p = 0.41', for Plaque score also.

## Discussion:

Dental caries is the third most common chronic non communicable disease that needs to be prevented and treated globally, according to the World Health Organization [W.H.O.] [10]. Dental caries pain negatively affects normal nutrition intake, speech, self-esteem, and daily routine activities, resulting in underweight children who experience abnormal cognitive development [11]. According to some studies, family-related risk factors such as parents level of education, family's income and employment status strongly influence the caries status of their children [12]. The use of DMFT/deft & Plaque indices has been an accepted practice for assessing the prevalence of caries and oral hygiene practice in a population. Hence an attempt has been made in the present study to assess the presence of dental caries and plaque in Siblings and to compare the role of family level factors such as parents' education, occupation, Family income, socioeconomic status and family size.

The study sample consisted of 232 Children of which 106 [45.7%] were males and 126 [54.3%] were females. Parents' age ranges from 27 to 49. 74% of the study population belongs to nuclear family. Educational status of family head revealed that majority of parents were educated, with 65.5% having completed schooling [primary, middle & high school], 34.5% were Diploma/Graduates and only 2.6% are Illiterate. About 60.3% of family head earned Rs 9232 - 27648 per month. Almost all parents were occupied in some form of occupation, from elementary work to Skilled Professionals.

In Kuppuswamy socio-economic status [SES] scale 2022 - almost 50.9% belonged to upper lower class which actually falls in low SES according to the Government of India in the National Family Health Survey [NFHS - II] using the Standard of Living Index [SLI] scale [8]. This can be attributed to the study being carried out in a government hospital [GCMCH]. The Government Chengalpattu Medical College Hospital [GCMCH] is the only governmental tertiary health care centre in Chengalpattu district and serves as the referral unit for several secondary care centres and Primary Health Centres [PHC's] in the area.

The overall prevalence of dental caries in the present study was found to be 61.6 %. Most of the contribution towards the dmft came from the decayed component, displaying urgent need for both reparative as well as preventive care in the population under consideration. These results were almost similar to studies reported by Misra *et al*. [13] [60.41%] and Chopra *et al*. [14] [61.88%]. However, many cross-sectional studies reported higher prevalence of dental caries. A study conducted in Bundelkhand region, India [15] found caries prevalence of 82.62% in a 3-14 years old group , A study by Rao *et al*. [16] found caries prevalence of 76.9% in 5-12-year-olds, and A study by Shingare *et al*. [17] reported prevalence of 80.92% among 3-14 year olds. A study by Sudha P *et al*. [18] reported prevalence of 82.5% in Mangalore city.

The above studies reported a much higher prevalence of dental caries compared to our study the reason for which might be due to difference in the social and the geographical locations studied. A very low caries prevalence of 18.01% was observed by Gupta DK *et al*. [19] among 6 to 12 year children which is attributed to the simple, traditional fibrous food consumed by children and good oral hygiene practices as reported by the author. Among gender, in the present study the female's DMFT/deft score was 69.8% and showed a slightly higher prevalence as compared to male children. This is similar to the findings by Misra *et al*. [13], Gaikwad *et al*. [20] and Singh *et al*. [21]. On the contrary, male children were found to have higher caries prevalence by Mahesh P *et al*. [22], Rao *et al*. [16], Sudha P *et al*. [18] and Auckland *et al*. [23]. This variation could be attributed to the difference in age groups studied. The status of presence of dental plaque in the present study was 48.3% this results was partly consistent with that of previous studies conducted in 2015 by Rafatjou R *et al*. [24] in Iran and study by Reddy MP *et al*. [25] Hyderabad, India in 2016. The presence of dental plaque was more in females [66.1%] than in males [33.9%] , which is similar to the finding by Reddy MP *et al*. [25] In present study the family structure, whether nuclear or joint, had no influence on the presence of dental plaque or caries experience of the child. This finding is similar to the study conducted by Sujlana *et al*. [26] in Haryana. One explanation for this finding could be that parents are the ones who devote time and energy to their children, regardless of the other family members. However, there is little information available regarding sibling relationships and the influence that siblings have on each other's attitudes about oral health.

The DMFT/deft value for first child is mean [M] = 2.64 & standard deviation [SD] = 3.55 which is lesser than the second/subsequent [average age is 5.63] [M = 3.27, SD =3.99]. Even though the average age of the first child is 8.9 which is almost 3 years more than the average age [5.63] of the second/subsequent. From the above results it is clear that even though the younger child with a lesser cariogenic exposure time than the elder ones developed more dental caries suggest elder child is strong predictors for caries development. There have been previous reports linking higher number of siblings to increased dental caries experience in younger children among siblings [27]. But this is exactly reverse in case of dental plaque score. Plaque scores for first child M = 0.74, SD =0.94 was greater than the second /subsequent child M = 0.65, SD =0.84. This is because the chance of developing dental plaque increased with age. But this difference is not statistically significant for both scores.

The parents' socioeconomic status was assessed by composite Kuppuswamy [8] SES scale which is based on parents' education, occupation of the head of the family, and total monthly family income. Parents' education level has been associated with the caries experience in their children [26]. In our study, the parents' level of education was found to be significantly associated with the dental plaque / caries experience in their children. A comparison of the level of education of parents revealed that the children of illiterate parents had an increased prevalence of both dental plaque and caries. The children of Illiterate parents were significant predictors of presence of dental plaque & caries. The relationship between lower parents education level and poor oral health status has also been reported in many previous studies [28]. Whereas, the difference between two groups of parents, one with primary, middle & high school education level and second with higher than high school education was not statistically significant. We evaluated the effect of the occupation of the parent on the dental plaque/caries experience of their children, which revealed a very significant relationship. Children of Machine operators / Assemblers, Craft and related trade workers had increased prevalence of caries whereas no significant relation existed between other occupations for caries prevalence. Children of Clerks, Skilled worker/ shop market sales workers were significantly associated with higher Dental plaque score, while no association was found between children of elementary occupation with that of dental plaque/caries experience. Because different studies used different classification criteria, the results of earlier studies are not comparable. However, research across the literature indicates that the parent's occupation has a discernible impact on the dental plaque and caries experiences of their offspring [28].

The various studies used a variety of proxy indicators for family income. Among these, over half indicated a negative correlation between dental caries and family income [28]. However, our study did not find any evidence of a relationship between dental caries experience and family income. Instead, it found a significant association between dental plaque presence and family income, particularly in the low monthly income group [less than 9226 per month]. This study illustrates a statistically significant relationship between socioeconomic status [SES] and the prevalence of dental plaque / caries. The association between SES and dental caries was unambiguous: most study that used a composite SES scale found that children from lower SES families had more dental caries than children from higher SES families [28]. In contrast to previous research, our study found no correlation between the lower middle and upper lower SES scale and a significant association between caries experience in the upper middle class, which might be due to increased frequency of dietary sugar intake and frequent snacking habit. In dental plaque assessment, the current study found no relationship in the upper middle class group and a significant relationship between dental plaque presence in lower middle and upper lower class groups. The most typical explanation for these phenomena is variations in oral hygiene practices. There are certain limitations to this study. It is a hospital based study and does not represent the general population. The results of the study's analysis are only applicable to the study population; they cannot be extrapolated to the entire population. It is also challenging to determine the direction of the established relationships between the variables in a cross-sectional study. To validate our findings, more longitudinal research is required.

## Conclusion:

The early years of a child's life establish the foundation for healthy lifestyle choices in adulthood. For children to establish good oral health that is dental plaque and caries free, it is crucial to evaluate family level factors such as parents' education, occupation, family income, socioeconomic status and family size. From the study, we can conclude that the family related factors such as parent's education, occupation, income and SES exerted a definite impact in the oral health of children than the family size and sibling factors.

## Figures and Tables

**Table 1:** Comparison of DMFT/deft and Dental plaque between different variables of respondents

	**Chi-Square test for > 0 DMFT/deft**			**Chi-Square test for dental plaque presence**		
	**Score**	**df**	**Sig.**	**Score**	**df**	**Sig.**
First & subsequent child	0.933	1	0.334	0.241	1	0.624
Gender	7.848	1	0.005	12.07	1	0.001
Family size	0.866	1	0.352	0.829	1	0.363
Education	10.111	2	0.006	10.984	2	0.004
Illiterate	9.896	1	0.002	6.599	1	0.01
Primary, middle & high school	1.256	1	0.262	2.252	1	0.133
Occupation	33.924	5	0	56.406	5	0
Clerks	0.667	1	0.414	39.771	1	0
Craft & related trade	9.756	1	0.002	0.02	1	0.886
Elementary Occupation	2.126	1	0.145	3.008	1	0.083
Machine operators/ assemblers	12.227	1	0	0.418	1	0.518
Skilled workers/ Agricultural/ Market sales	1.891	1	0.169	17.188	1	0
Family Income	2.57	3	0.463	21.845	3	0
>9226	1.533	1	0.216	0.032	1	0.858
9232 to 27648	1.688	1	0.194	5.107	1	0.024
27654 to 45089	0.092	1	0.762	15.134	1	0
46095 to 68961	0.475	1	0.491	8.878	1	0.003
SES Scale	12.108	3	0.007	7.935	3	0.047
Upper	1.533	1	0.216	0.032	1	0.858
Upper middle	9.756	1	0.002	0.02	1	0.886
Lower middle	0.963	1	0.326	7.514	1	0.006
Upper lower	2.024	1	0.155	5.637	1	0.018
NS: Non-significance, * < 0.05 = significance.

**Table 2:** The Mean & Standard Deviation values of DMFT/deft, Plaque scores in the first and second /subsequent child

		**DMFT/deft SCORE**	**PLAQUE SCORE**
First child	No. of child	108	108
	Mean	2.64	0.74
	Std. Deviation	3.553	0.941
Second & subsequent child	No. of child	124	124
	Mean	3.27	0.65
	Std. Deviation	3.994	0.838
Total	No. of child	232	232
	Mean	2.97	0.69
	Std. Deviation	3.8	0.887

**Table 3:** Results of ANOVA comparing the DMFT/deft Score, Dental plaque score with first and second/subsequent child.

**ANOVA**						
		**Sum of Squares**	**df**	**Mean Square**	**F**	**Sig.**
DMFT	Between Groups	22.71	1	22.71	1.577	.211 (NS)
	Within Groups	3313.134	230	14.405		
	Total	3335.845	231			
PLAQUE SCORE	Between Groups	0.527	1	0.527	0.67	.414 (NS)
	Within Groups	181.128	230	0.788		
	Total	181.655	231			
NS: Non-significance, * < 0.05 = significance.

**Table 4:** Comparison of mean deft/DMFT and Plaque score with socioeconomic status which was found to be significant statistically (P < 0.05) using one way ANOVA test.

**ANOVA**						
		**Sum of Squares**	**df**	**Mean Square**	**F**	**Sig.**
DMFT	Between Groups	181.555	3	60.518	4.374	.005*
	Within Groups	3154.29	228	13.835		
	Total	3335.845	231			
PLAQUE SCORE	Between Groups	14.96	3	4.987	6.82	.000*
	Within Groups	166.695	228	0.731		
	Total	181.655	231			
*< 0.05 = significance.

## References

[1] Bennadi D (2014). Asian J Med Sci..

[2] Singh M (2010). J Indian Assoc Public Health Dent..

[3] Baginka J, Rodakowska E. (2012). Int J Collab Res Intern Med Public Health..

[4] Wen A (2017). JDR Clin Transl Res..

[5] Mansbridge JN (1959). J Dent Res..

[6] Shearer DM, Thomson WM. (2010). Community Dent Oral Epidemiol..

[7] Dobloug A, Grytten J. (2016). Community Dent Oral Epidemiol..

[8] Kumar G (2022). Int J Community Dent..

[9] https://www.who.int/publications/i/item/9789241548649.

[10] Marrs JA (2011). Pediatr Nurs..

[11] Grewal H (2009). J Indian Soc Pedod Prev Dent..

[12] Stephen A (2015). J Int Soc Prev Community Dent..

[13] Misra FM, Shee BK. (1979). J Indian Dent Assoc..

[14] Chopra S (1983). J IndianDent Assoc..

[15] Jain A (2016). Int J Community Med Public Health.

[16] Rao A (1999). J Indian Soc Pedod Prev Dent..

[17] Shingare P (2012). J Contemp Dent.

[18] Sudha P (2005). J Indian Soc Pedod Prev Dent..

[19] Gupta DK (2016). Prog Orthod..

[20] Gaikwad RS, Indurkar MS. (1993). J Indian Dent Assoc..

[21] Singh M (2011). Indian J Dent Res..

[22] Kumar PM (2005). J Indian SocPedodPrev Dent Mar..

[23] Aukland S, Bjelkaroey J. (1982). J Indian Dent Assoc..

[24] Rafatjou R (2016). Journal of dental. Medicine..

[25] Reddy MP (2016). Journal of Indian Association of Public Health Dentistry..

[26] Sujlana A, Pannu P. (2015). J Indian Soc Pedod Prev Dent..

[27] Paula JS (2012). Health Qual Life Outcomes..

[28] Kumar S (2016). J Dent..

